# A Role for S1P and S1P1 in Immature-B Cell Egress from Mouse Bone Marrow

**DOI:** 10.1371/journal.pone.0009277

**Published:** 2010-02-18

**Authors:** João Pedro Pereira, Jason G. Cyster, Ying Xu

**Affiliations:** Department of Microbiology and Immunology, University of California San Francisco, San Francisco, California, United States of America; New York University, United States of America

## Abstract

B lymphocyte egress from secondary lymphoid organs requires sphingosine-1-phosphate (S1P) and S1P receptor-1 (S1P1). However, whether S1P contributes to immature-B cell egress from the bone marrow (BM) has not been fully assessed. Here we report that in S1P- and S1P1-conditionally deficient mice, the number of immature-B cells in the BM parenchyma increased, while it decreased in the blood. Moreover, a slower rate of bromodeoxyuridine incorporation suggested immature-B cells spent longer in the BM of mice in which S1P1-S1P signaling was genetically or pharmacologically impaired. Transgenic expression of S1P1 in developing B cells was sufficient to mobilize pro- and pre-B cells from the BM. We conclude that the S1P1-S1P pathway contributes to egress of immature-B cells from BM, and that this mechanism is partially redundant with other undefined pathways.

## Introduction

In late stages of B cell development in the bone marrow (BM), newly generated immature-B cells become partitioned between the BM parenchyma and sinusoids [1], [2], [3]. Immature-B cells are retained in the sinusoidal compartment for a short period before entering the blood circulation [3]. Sinusoidal entry is thought to be a key step in egress commitment yet the requirements for this migration event remain poorly defined.

Egress of lymphocytes from the thymus and from peripheral lymphoid organs requires a concentration gradient of Sphingosine 1-Phosphate (S1P) [4], [5]. S1P is recognized by S1P receptor-1 (S1P1) expressed on lymphocytes, and deficiency in S1P1 results in a severe egress block from the thymus and lymph nodes [6]. The small molecule FTY720 functionally antagonizes the activity of S1P1, blocks lymphocyte egress from secondary lymphoid organs, and causes peripheral lymphopenia [6], [7], [8]. Despite this major role in egress, deficiencies in S1P or S1P1 did not reveal a dominant role for this ligand-receptor system during B cell egress from the BM [4], [6]. In short-term adoptive transfer experiments S1P1-deficient mature B cells accumulated in the BM, but a role for S1P1 in egress was not established [9]. Recently, NK cells were shown to require intrinsic S1P5 expression for BM egress and conditional deficiency in both S1P-generating enzymes, Sphingosine kinase (Sphk)-1 and -2, reduced NK cell egress from the BM [10], [11]. Monocyte egress from the BM has been suggested to be promoted by S1P receptor agonist treatment though a direct effect of S1P receptor or S1P deficiency on monocyte egress has not yet been demonstrated [12].

Here we show that in mice conditionally deficient in S1P1 in B-lineage cells, egress of immature-B lymphocytes from the BM was slightly but significantly reduced. In S1P-deficient mice immature-B cell egress was also diminished. Reciprocally, premature expression of S1P1 from a transgene was sufficient to mobilize pro- and pre-B cells into the periphery. These findings indicate that the S1P-pathway contributes to the mechanism of B cell egress from the BM.

## Materials and Methods

### Mice, Chimeras, In Vivo FTY720 Treatments, and BrdU Labeling

Adult C57Bl/6 (Ly5.2^+^) mice aged 6–8 weeks were from the National Cancer Institute, and adult Boy/J (Ly5.1^+^; stock no. 002014) mice were from The Jackson Laboratories. *Edg1^fl/fl^* and *Edg1^+/−^* mice (obtained from Dr. Richard Proia, National Institute of Diabetes and Digestive and Kidney Diseases, Bethesda, MD) were crossed with *Mb1^Cre/+^* [13] (Dr. M. Reth, Max-Planck Institute of Immunobiology, Freiburg, Germany) to generate *Edg1^fl/−^Mb1^Cre/+^* and *Edg1^+/+^Mb1^Cre/+^*. *Sphk1^fl/−^ Sphk2^−/−^* mice [4] were provided by Dr. Shaun Coughlin (University of California San Francisco, CA) and carried an Mx-Cre transgene [14]. BM chimeras were prepared as described [3], and analyzed at least 6 weeks after reconstitution. FTY720 was from a custom synthesis by Stanford Resesarch Institute (Palo Alto, CA). Adult C57Bl/6 mice were treated with FTY720 at 1 mg/Kg (or saline) intravenously (i.v.) for 3 h or 3 days. BrdU labeling experiments were as described [3]. Animals were housed in a specific pathogen-free facility, and all experiments were performed in accordance with protocols approved by the University of California San Francisco Institutional Animal Care and Use Committee.

### Generation of S1P1 Transgenic Mice

A DNA fragment encoding mouse *Edg1* mRNA was PCR amplified from pMSCV-Flag-Edg1, with the following primers: forward 5′- GTG GAT CCC CCG GGC TGC AGG AGT T -3′ (containing a 5′ BamHI restriction site) and reverse 5′- CAT GCT CGA GTT ATT AGG AAG AAG AA -3′ (containing a XhoI restriction site). The PCR product was digested with BamHI and XhoI restriction enzymes (Roche) and cloned into the plasmid p1026x containing the immunoglobulin Eμ heavy chain enhancer and the Lck proximal promoter [15]. The NotI linearized plasmid was microinjected into fertilized (C57BL/6 x DBA/2J) oocytes according to standard procedures. Transgenic mice (line D) were screened by PCR using the primers described below. All the transgenic mice analyzed were heterozygous for the transgene, and segregated at the expected Mendelian rate. All mice were healthy at all ages tested.

### In Vivo Labeling of Bone Marrow Sinusoidal B Cells and In Vitro Migration Assays

Sinusoidal B cells were labeled as described [3]. In FTY720, and BrdU treatments, *in vivo* labeling was performed in the last 2 minutes of the indicated treatments. Developing B cell subsets were gated according to Hardy's nomenclature [16], with modifications, as described [3]. Briefly, pro-B and pre-B cells were gated as forward scatter large and small B220^+^ (PE-Cy5.5 conjugated clone RA3-6B2, Biolegend), CD93^+^ (APC conjugated clone AA4.1, eBioscience), IgM^−^ (FITC conjugated Goat anti mouse IgM, Jackson Immunoresearch) and IgD^−^ (biotin-conjugated Rat anti mouse IgD, clone 11–26, Southern Biotech.), revealed with streptavidin QD605, (Molecular Probes) lymphocytes, respectively; immature-B cells were divided into two populations: B220^+^CD93^+^IgM^+^IgD^−^ and B220^+^CD93^+^IgM^+^IgD^lo^; mature recirculating B cells were isolated as B220^+^CD93^−^IgM^+^IgD^hi^ cells. Chemotaxis assays to S1P (Sigma) or CXCL12 (R&D) were performed as described [17].

### Cell Sorting and mRNA Analyses

BM cells were stained at 40×10^6^ cells/mL, and B cell subsets were identified as described. Cells were sorted with a BD FACS ARIA instrument, and the purity of sorted populations was >97%. Quantitative PCR (ABI 7700 PE Applied Biosystems) was performed with the following primers/probes (Integrated DNA Technologies): *Edg1* forward 5′- ccttcatccggatcgtatct -3′, and reverse 5′- tgctgcggctaaattccatg -3′. *Edg1* amplification was as follows: 35 cycles 94°C for 20 s; 55°C for 20 s; and 72°C for 30 s; other primers used were as described [3].

## Results

### Reduced BM Egress of S1P1-Deficient Immature-B Cells

**Figure 1:**
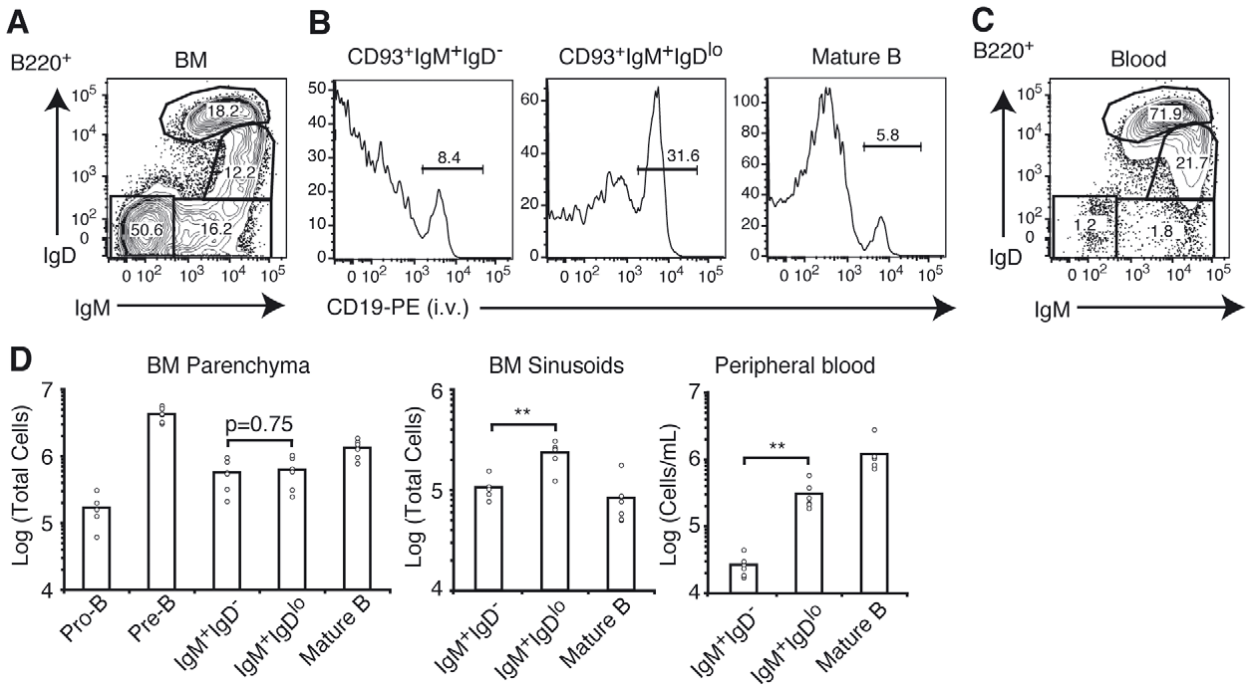
Progression into the Immature IgD^lo^ B cell stage coincides with increased egress efficiency. (A) Expression of IgM and IgD in B220^+^ BM B-lineage cells. Numbers indicate percent of total B220^+^ cells. (B) Distribution of the indicated B-lineage populations in BM parenchyma and sinusoids. Numbers indicate percent of cells from each subset residing in sinusoids. (C) Expression of IgM and IgD in B220^+^ B-lineage cells in peripheral blood. Numbers indicate percent of total B220^+^ cells. (D) Enumeration of developing B cell subsets in BM parenchyma and sinusoids, and peripheral blood. In all panels, data shown are representative of more than 10 independent experiments. ** P<0.005 (unpaired, two-tailed Student's *t*-test).

In adult wild type mice, two subsets of BM immature CD93^+^IgM^+^ B cells can be distinguished based on surface IgD expression (Figure 1A). To distinguish by flow cytometry B cell subsets that were in the BM parenchyma, or in sinusoids, we injected 1 µg of phycoerythrin (PE)-conjugated anti-CD19 intravenously and allowed the antibody to equilibrate for 2 minutes, an exposure period that achieves selective labeling of sinusoidal B cells [3]. Whereas the number of immature CD93^+^IgM^+^IgD^−^ and CD93^+^IgM^+^IgD^lo^ cells was similar in the BM parenchyma (Figure 1B and D), the IgD^lo^ subset was more abundant in BM sinusoids (Fig. 1B and D), and approximately 10-fold enriched in the peripheral blood (Figure 1C and D). This observation suggests that progression into the IgD^lo^ stage coincides with a higher propensity of these cells to egress from the BM parenchyma into sinusoids, and then into circulation.

**Figure 2:**
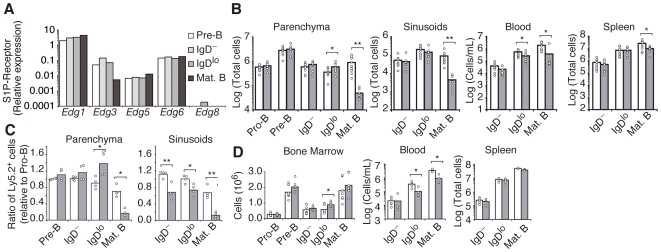
Impaired BM egress of S1P1-deficient immature-B cells. (A) Quantitative RT-PCR analysis of transcript abundance for the genes encoding S1P1 (*Edg1*), S1P2 (*Edg5*), S1P3 (*Edg3*), S1P4 (*Edg6*) and S1P5 (*Edg8*) in purified developing BM B cell subsets presented relative to *Hprt1*. Data shown are representative of 3 experiments. (B) Number of B cells in indicated subsets in the BM parenchyma and sinusoids, peripheral blood and spleens of *Edg1^Fl/−^ Mb1^Cre/+^* (gray bars) and *Edg1^+/+^ Mb1^Cre/+^* (white bars) mice. Data are pooled from 4 independent experiments. (C) Analysis of B cell development in chimeras reconstituted with mixtures of *Edg1^Fl/−^ Mb1^Cre/+^* (gray bars, Ly5.2^+^) or *Edg1^+/+^ Mb1^Cre/+^* (white bars, Ly5.2^+^) and wild-type (Ly5.1^+^) BM. Shown is the ratio of Ly5.2^+^ cells in the indicated developmental subsets with that of Pro-B cells in the same animal. Data are pooled from 3 independent experiments. (D) Number of developing B cells in total BM, blood and spleens of mice treated with FTY720 (1 mg/Kg, gray bars) or with saline (white bars) for 3 h. Data shown are representative of more than 3 independent experiments. * P<0.05; ** P<0.005 (unpaired, two-tailed Student's *t*-test).

Analysis of S1P receptor-encoding transcript abundance in sorted BM B cell populations revealed S1P1 to be the most abundant receptor, already expressed in pre-B cells (Figure 2A). Expression of S1P2, S1P3 and S1P4 was also detected, whereas S1P5 was undetectable (Figure 2A). To assess if S1P1 plays a role in immature-B cell egress from the BM we analyzed the distribution of developing B cells in BM parenchyma and sinusoids, peripheral blood and spleens of mice in which S1P1 was conditionally ablated in B-lineage cells (hereafter designated as S1P1-deficient). In S1P1-deficient mice, the number of immature IgD^lo^ B cells was slightly, but significantly, increased in the BM parenchyma, and reduced by 2.1-fold in peripheral blood (Figure 2B). This difference contrasted with similar numbers of BM parenchymal Pro-B, Pre-B and immature IgD^−^ B cells, thus suggesting that BM egress of immature IgD^lo^ cells might be specifically reduced in S1P1-deficient mice. The number of immature IgD^lo^ cells in sinusoids seemed, however, unaffected (Figure 2B). Mature B cells were significantly reduced in BM, blood and spleen (Figure 2B), as previously reported [6]. Given that egress of hematopoietic cells from the BM is thought to occur via BM sinusoids, it was surprising to find that while S1P1-deficient immature-IgD^lo^ cells accumulated in parenchyma, their numbers seemed unaffected in sinusoids. However, the absolute cell numbers present in BM sinusoids are small and variable between mice (Figure 2B). To facilitate detection of even small changes in cell representation in sinusoids, we turned to a mixed BM chimera approach that would allow comparison of wildtype and S1P1-deficient cell frequencies in sinusoids of the same animals. We analyzed the distribution of B cell subsets in BM parenchyma and sinusoids of mice reconstituted with mixtures of *Edg1^fl/−^ Mb1^Cre/+^* or *Edg1^+/+^ Mb1^Cre/+^* (Ly5.2) and wild type (Ly5.1) BM. We found an accumulation of S1P1-deficient immature IgD^lo^ B cells in the BM parenchyma (Figure 2C), consistent with the above findings (Figure 2B). Moreover, by this approach we found that the fraction of S1P1-deficient immature IgD^−^ and IgD^lo^ B cells was reduced in sinusoids (Figure 2C), suggesting that egress from BM parenchyma into sinusoids was reduced.

As an approach to test whether S1P signaling acts continually to promote immature-B cell egress from BM we treated C57BL/6 mice with FTY720 (1 mg/Kg). After a 3 h treatment there was a significant accumulation of immature IgD^lo^ cells in BM, and a 3-fold reduction in peripheral blood (Figure 2D), consistent with a reduced rate of BM egress and recirculation.

### Reduced Egress of Immature-B cells from BM of S1P-Deficient Mice

**Figure 3:**
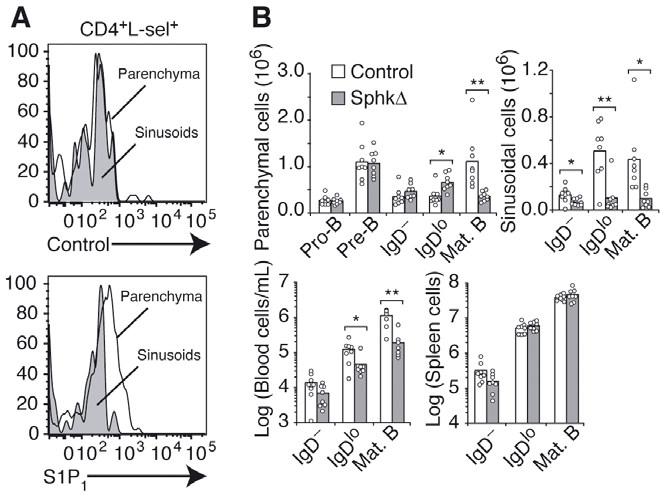
S1P1 surface expression in BM parenchymal and sinusoidal cells and impaired egress of immature-B cells from the BM of S1P-deficient mice. (A) S1P1 surface expression in BM parenchymal and sinusoidal naïve CD4^+^ T cells. Mice were treated with Ly5.2-PE antibodies intravenously for 2 minutes. T cells were gated as TCRβ^+^ CD4^+^ CD8^−^ L-sel^+^, and further gated as parenchymal (Ly5.2^−^, black) and sinusoidal (Ly5.2^+^, gray). Top panel shows cells stained with a control rabbit antiserum, and bottom panel shows cell staining with an anti-S1P1 rabbit antiserum. Data are representative of two experiments (n = 6). (B) Number of developing B cell subsets in the BM parenchyma, sinusoids, blood and spleen of *Sphk-1* and *-2*-deficient and littermate control mice. Data shown were pooled from 4 independent experiments. * P<0.05; ** P<0.005 (unpaired, two-tailed Student's *t*-test).

S1P1 is sensitive to down-modulation by ligand, and surface levels on CD4 T cells are high when cells are in low S1P environments and low when cells are in high S1P environments [4], [17]. Flow cytometric analysis of BM parenchymal and sinusoidal naïve CD4^+^ T cells showed detectable surface S1P1 on parenchymal cells compared to a lack of surface receptor on sinusoidal cells (Figure 3A) as recently reported [10], indicating that S1P concentrations are lower in the parenchyma than in sinusoids. Mice conditionally deficient in *Sphk1* and *Sphk2* (*Sphk*-deficient) showed normal numbers of developing B cells in BM, but their distribution was not assessed [4]. Thus, we revisited B cell development in *Sphk*-deficient mice, analyzing positioning of B cell subsets in BM by flow cytometry. We found a small but significant increase in the number of parenchymal immature IgD^lo^ cells, and a reduction in their numbers within sinusoids (Figure 3). The number of immature IgD^lo^ cells was approximately 2.6-fold lower in peripheral blood of S1P-deficient mice than in littermate controls. The accumulation in BM parenchyma and reduction in peripheral blood suggests that S1P also contributes to immature-B cell egress from BM (Figure 3).

**Figure 4:**
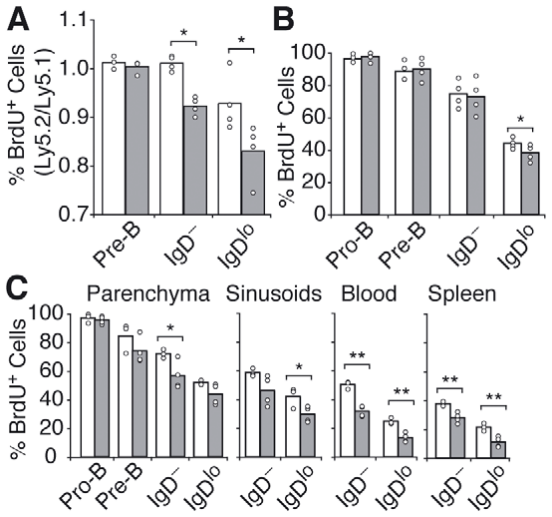
Prolonged residence of immature-B cells in BM of S1P1 or S1P deficient mice. (A) Analysis of BrdU incorporation for 48 h in developing B cell subsets in chimeras reconstituted with mixtures of *Edg1^Fl/−^ Mb1^Cre/+^* (gray bars, Ly5.2^+^) or *Edg1^+/+^ Mb1^Cre/+^* (white bars, Ly5.2^+^) with wild-type (Ly5.1^+^) BM. Shown is the ratio of percent BrdU^+^ Ly5.2^+^ and BrdU^+^ Ly5.1^+^ cells in the indicated developmental subsets. Data are pooled from 3 independent experiments. (B) BrdU incorporation for 48 h in developing B cell subsets from *Sphk-1* and *-2*-deficient (gray bars) and littermate controls (white bars). Data shown were pooled from 2 independent experiments. (C) BrdU incorporation for 48 h in developing B cell subsets from mice treated with saline (white bars) or FTY720 (1 mg/Kg; gray bars) for 3 days. Data shown are representative of 3 independent experiments. In all panels, bars indicate the mean, circles indicate individual mice. * P<0.05; ** P<0.005 (unpaired, two-tailed Student's *t*-test).

If BM egress of developing B cells is defective then their time of residence in the BM might be increased. To explore this possibility we analyzed the rate of BrdU incorporation in BM B cell subsets of *Edg1^fl/−^ Mb1^Cre/+^* or *Edg1^+/+^ Mb1^Cre/+^* (Ly5.1) and wild type (Ly5.2) mixed BM chimeras, and in *Sphk*-deficient and control littermates, by feeding them BrdU (1 mg/mL) in the drinking water. After 48 hours, the fraction of BrdU^+^ S1P1-deficient immature IgD^−^ and IgD^lo^ B cells was significantly reduced, as compared to the fraction of BrdU^+^ S1P1-sufficient immature-B cells (Figure 4A). Pre-B cells became similarly BrdU labeled in the two groups indicating that S1P1-signaling is not required for Pro-B and Pre-B cell expansion or survival. Thus, the reduced frequency of BrdU^+^ immature-B cells likely reflects an increase in the proportion of these cells that resided in BM for a period longer than the 48 h of continuous BrdU treatment. *Sphk*-deficient mice also showed lower 48 h BrdU labeling of IgD^lo^ immature-B cells compared to littermate controls (Figure 4B). Similarly, in wild-type C57BL/6 mice treated with FTY720 for 3 days we found a reduced fraction of 24 h and 48 h BrdU-labeled immature-B cells (but not Pre-B cells) in BM parenchyma, sinusoids, blood and spleen (Table 1 and Figure 4C). The similar rate of BrdU incorporation at 24 h and 48 h in Pro-B and Pre-B cells suggests that the S1P/S1P-receptor pathway is not required during early stages of B cell development. However, these findings suggest a slower rate of immature-BM B cell replacement by newly generated cells, consistent with a role for the S1P-pathway in BM egress.

**Table 1:** Frequency of BrdU^+^ cells in the indicated BM B cell developmental subsets from mice treated with saline or FTY720 (1 mg/Kg) for 3 days, and continuously exposed to BrdU in the last 24 h.

*Cell Subset	Pro-B	Pre-B	IgM^+^IgD^−^	IgM^+^IgD^lo^	Mature-B
Saline	88.5±6.5	63.7±5.2	31.8±1.6	16.3±1.8	0.2±0.1
FTY720	81.0±9.3	62.2±3.8	21.4±7.5	12.9±3.3	0.2±0.1
Student's *t*	0.29	0.67	0.02	0.07	0.70

* Immature B cell subsets (IgM^+^IgD^−^ and IgM^+^IgD^lo^) were gated as CD93^+^. Values indicate Average ± SD of pooled data from 2 independent experiments (n = 4 in each group).

### Premature Egress of Pro- and Pre-B Cells in S1P1-Transgenic Mice

**Figure 5:**
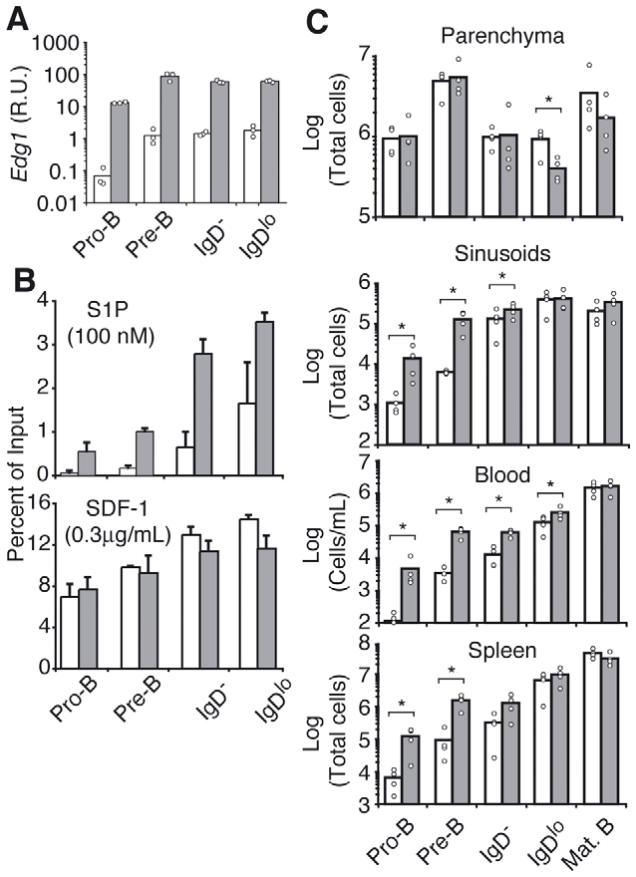
Premature egress of B cell precursors from the BM of S1P1-transgenic mice. (A) Quantitative RT-PCR analysis of the expression of S1P1 encoding mRNA in purified developing BM B cell subsets from S1P1-transgenic (gray bars) and littermate control (white bars) mice, presented relative to *Hprt1* mRNA (hypoxanthine guanine phosphoribosyl transferase). Data shown was pooled from 3 independent experiments. (B) Migration assays of developing B cells from S1P1-transgenic (gray bars) and littermate control (white bars) mice to S1P (100 nM) or SDF-1 (0.3 µg/mL). Data are representative of 4 experiments. (C) Enumeration of developing B cell subsets from S1P1-transgenic (gray bars) and littermate control (white bars) mice in the BM parenchyma, sinusoids, peripheral blood and spleen. In all panels, bars indicate the mean, circles indicate individual mice. * P<0.05; ** P<0.005 (unpaired, two-tailed Student's *t*-test).

Finally, we asked if enforced expression of S1P1 in B lymphocytes was sufficient to promote premature egress of developing B cells. We generated transgenic mice expressing S1P1 under the control of the immunoglobulin heavy chain *Eμ* enhancer and *Lck* proximal promoter, and analyzed S1P1 expression during B cell development. S1P1 transcripts were significantly increased already at the Pro-B cell stage and remained higher in transgenic B cell subsets than in cells from littermate controls (Figure 5A). Moreover, *in vitro* chemotaxis assays showed increased migration of S1P1-transgenic Pro-B, Pre-B, and immature-B cells to S1P, indicating that S1P1 is functional in these cells (Figure 5B). Migration of B cell subsets from S1P1-transgenic and littermate controls to SDF-1 was similar (Figure 5B). Analysis of developing B cell numbers showed a 10-fold increase in Pro- and Pre-B cells in sinusoids, peripheral blood and spleens of S1P1-transgenic mice (Figure 5C). Immature-B cell distribution was altered as well, with reduced numbers in the BM parenchyma and increased numbers in sinusoids, blood and spleen (Figure 5C). Even though Pro- and Pre-B cells were substantially increased in sinusoids, blood and spleen, the total number of cells detected in the periphery was in the order of 10^4^–10^5^ and thus insufficient to cause a measurable decrease from the parenchyma (where the numbers are in the order of 10^6^).

## Discussion

Several studies have demonstrated that the S1P-S1P1 pathway operates to control lymphocyte egress from primary and secondary lymphoid organs [18]. However, these studies did not reveal a role for this pathway in immature-B cell egress from the BM. Egress of BM cells is thought to occur via the vast network of sinusoids that travels through the parenchyma [19]. By using a pulse-labeling procedure that specifically labels cells residing in the BM vasculature [3], we assessed if the S1P-S1P1 pathway contributes to the immature-B cell distribution between BM parenchyma and sinusoids. We showed that in mice conditionally deficient in S1P1 in B lineage cells, and in mice in which S1P1 activity was pharmacologically ablated, immature-B cells accumulated in the BM parenchyma and were concomitantly reduced in sinusoids and peripheral blood, suggesting reduced egress efficiency. S1P-deficient mice also exhibited reduced egress of immature-B cells from the BM. Reciprocally, transgenic expression of S1P1 early in B cell development resulted in the mobilization of Pro-B and Pre-B cells from the BM parenchyma into sinusoids, peripheral blood and spleen.

Our finding of a reduction in 48 hr BrdU-labeled S1P1- and S1P-deficient immature-BM B cells compared to wildtype controls indicated that it takes longer to fully replace S1P1- and S1P-deficient immature cells with cells newly generated from pre-B cells. Since the animals showed similar extents of pre-B cell labeling at 24 h and 48 h, we feel that the simplest interpretation of these data is that the immature-B cells reside in the BM for a slightly longer average time due to less efficient egress. We cannot exclude the possibility that S1P1 and S1P-deficiency somehow influence other properties of the immature-B cells, such as reducing the fraction that undergo cell death. However, S1P1 signaling has been found to promote improved cell viability in some systems [20], [21], [22], making this explanation seem unlikely.

It was notable that S1P-deficient mice showed a greater reduction in the sinusoidal immature-B cell population than observed in S1P1-deficient mice. This result could be explained by a contribution of additional S1P receptors in egress of immature-B cells from BM. S1P3 is differentially expressed in developing B cell subsets, being most abundant in immature IgD^−^ and IgD^lo^ cells, and *in vitro* chemotactic responsiveness of immature-B cells to S1P is predominantly S1P3-mediated, indicating the receptor is functional in these cells [23], [24] (and data not shown). Indeed, as for marginal zone B cells [25], the activity of S1P3 in the *in vitro* S1P chemotaxis assay obscures the activity of S1P1. However, analysis of B cell development in S1P3-deficient mixed BM chimeras (n = 10) revealed no significant differences in immature-B cell BM egress (data not shown). A recent study also found a lack of S1P3 contribution to immature B cell egress from the BM [24]. The basis for the discord between S1P receptor activity *in vitro* versus *in vivo* is unclear but might reflect the high sensitivity of S1P1 to desensitization [17], [26], [27] and the lack of appropriate partioning of S1P distribution in the *in vitro* assay. S1P4 transcripts were also detected in immature-B cells and it is possible that this poorly characterized receptor contributes to the egress mechanism. Alternatively, S1P may have a B cell extrinsic as well as S1P1-dependent B cell intrinsic effect. For example, endothelial cells can respond to S1P [28] and S1P-deficiency might lead to alterations in sinusoidal endothelium that impair retention of immature and mature B cells. 2-arachydonoyl glycerol (2-AG) can be produced by endothelial cells [29], [30], [31], is abundant in the BM microenvironment [3], and its receptor, cannabinoid receptor 2 (CB2), is required for B cell retention in sinusoids [3]. It will be important in future studies to determine the 2-AG concentration in BM of *Sphk*-deficient mice.

Although S1P1 is already present in pre-B cells, they undergo minimal egress compared to immature B cells. Concordantly, in S1P1-transgenic mice S1P1 transcripts are equivalently high in Pre-B and Immature B cells, yet the immature B cell population has higher egress efficiency as measured by numerical reduction in BM parenchyma and increase in sinusoids, peripheral blood and spleen. Thus, we propose that during B cell development additional changes occur that confer an increased ability to egress the BM. These changes may be related to alterations in BM parenchyma retention, expression of additional egress receptor(s), or both.

In summary, we suggest that immature-B cell movement from parenchyma into sinusoid is promoted by S1P1-signaling within the B cell in response to the higher S1P concentration within the sinusoid. The partial nature of the S1P-pathway contribution to B cell egress from the BM likely reflects redundancy with additional mechanisms such as down-regulation of CXCR4-mediated retention [32], [33] and expression of further receptors sensing sinusoid associated or circulatory chemoattractants.
