# Correction: A Role for S1P and S1P1 in Immature-B Cell Egress from Mouse Bone Marrow

**DOI:** 10.1371/annotation/2ae645ec-9413-4f7d-b51f-eb0678fa2f1b

**Published:** 2010-03-04

**Authors:** João Pedro Pereira, Ying Xu, Jason G. Cyster

The order of the second and third authors was reversed. Ying Xu should be the second author and Jason G. Cyster should be the third author. The correct citation is: Pereira JP, Xu Y, Cyster JG (2010) A Role for S1P and S1P1 in Immature-B Cell Egress from Mouse Bone Marrow. PLoS ONE 5(2): e9277. doi:10.1371/journal.pone.0009277 

